# Dr. W. H. Morgan

**Published:** 1901-06-15

**Authors:** 


### OBITUARY.

### DR. W. H. MORGAN.

Dr. W. H. Morgan, of Nashville, Tenn., died May 16th at his home in Nashville, in the eighty-fourth year of his life. His death removes another man from our profession, who, in the active years of his life, exerted a great influence in dignifying his calling by devoting all his great mental strength to enlarging others' conceptions of its great usefulness by personally demonstrating it in his own practice. He was not only a leader technically but he was scholarly, and did much to advance the standards of education. He believed that education was as essential for the dental as for the medical profession. He was highly esteemed by the profession not only of the South, but he was well known all through the North and East, and was always given a most respectful hearing when he arose to speak in our national societies. He was a man of strong convictions, liberal, and deeply religious. He held many high positions of trust in civil and national government and in the Methodist Church as well as in the Masonic fraternity. He has been honored as presiding officer of many National and State Dental Associations. He graduated from the Baltimore College of Dental Surgery in 1848, and began practice in Nashville in 1849. He became a trustee of the Ohio Dental College in 1865, and continued an active member of this board until 1879, when he organized the Dental Department of Vanderbilt University, and acted as its dean until a few years ago, when, because of illness, he was compelled to resign the chair. It was our good fortune to know Dr. Morgan while in college, and we have kept more or less in contact with him ever since. He was kind and genial and always ready to give counsel to the young, and his life was wholly consecrated to doing everything he thought would bring the Divine kingdom into the hearts of those whom he could influence. His presence among us will be missed, but the precious memory of his noble life is a blessing to be always cherished with affectionate admiration.

